# Clinical Significance of Isolated Third Cranial Nerve Palsy in Traumatic Brain Injury: A Detailed Description of Four Different Mechanisms of Injury through the Analysis of Our Case Series and Review of the Literature

**DOI:** 10.1155/2021/5550371

**Published:** 2021-04-23

**Authors:** Micaela Uberti, Shumaila Hasan, David Holmes, Mario Ganau, Chris Uff

**Affiliations:** ^1^Department of Neurosurgery, The Royal London Hospital, London E1 1FR, UK; ^2^Department of Neurosurgery, Oxford University Hospitals, Oxford OX3 9DU, UK; ^3^Centre for Trauma Sciences, Queen Mary University of London, London E1 4NS, UK

## Abstract

Third cranial nerve palsy (3cnP) following traumatic brain injury (TBI) is a worrying neurological sign and is often associated with an expanding mass lesion, such as extradural or acute subdural haematomas. Isolated 3cnP can be found in the absence of posttraumatic space-occupying mass lesion, yet it is often considered as a devastating prognostic factor in the context of diffuse axonal injury (DAI). Through the analysis of five exemplificative cases and a thorough review of the literature, we identified four possible mechanisms leading to 3cnP: (1) a partial rootlet avulsion at the site of exit from the midbrain, representing a direct shearing injury to the nerve; (2) a direct traction injury due to the nerve stretching against the posterior petroclinoid ligament at the base of the oculomotor triangle secondary to the downward displacement of the brainstem at the time of impact; (3) a direct vascular compression as a result of internal carotid artery (ICA) dissection or pseudoaneurysm; (4) an indirect injury caused by impaired blood supply to the third nerve in addition to the detrimental biochemical effects of the underlying brain injury itself. Understanding the exact mechanism underlying the onset of 3cnP is key to provide an informed clinical decision-making to the patients and ensure their best chances of recovery. Our experience corroborates data from the literature showing that, even in Grade III DAI, prompt recognition of isolated 3cnP can guide adequate treatment. Nonetheless, even when an overall good neurological outcome is achieved, recovery of isolated 3cnP is dismal, and only rarely the visual deficit completely resolves.

## 1. Introduction

Third cranial nerve (oculomotor) palsy (3cnP) is seen in all grades of traumatic brain injury (TBI), either immediately after the traumatic event or evolving hours to days after it [1]. 3cnP represents a worrying neurological sign because it is often associated with an expanding mass lesion, such as extradural or subdural haematomas [2, 3]. Isolated 3cnP can be found in the absence of posttraumatic space-occupying mass lesion, yet it is often considered as a devastating prognostic factor in the context of diffuse axonal injury (DAI).

Isolated 3cnP is very rare; its incidence ranges from 8 to 16%, depending on the clinical series [4–9]. In terms of clinical presentation, it can be found in combination with other cranial nerve deficits (particularly VI, V, and VI), can be unilateral or bilateral, and can be transient or permanent [9].

Unfortunately, the pathological basis of isolated 3cnP has been poorly described in previous reports. An oculomotor injury can be the consequence of lesions located anywhere from the oculomotor nucleus in the midbrain to the termination of the third cranial nerve in the extraocular muscles within the orbit. The size of the pupil and its reaction to light can indicate where the lesion is most likely to be located [4, 10]. In the early stages of central herniation, there is a diffuse bilateral hemisphere dysfunction due to decreased blood flow from increased intracranial pressure (ICP) and diencephalic dysfunction due to downward displacement. In this stage, pupils are normally small (1–3 mm) and reactive to light. In later stages, when the midbrain is affected, pupils are moderately dilated (3–5 mm) and fixed. This stage is also seen in uncal herniations. During the terminal stage of herniation, where the medulla is affected, the diffuse hypoxia continues to sustain mydriasis; therefore, the damage becomes irreversible. Fixed dilated pupils have thus historically been associated with a very poor prognosis in TBI [11–15].

Imaging alone can be ambiguous in defining the management. CT scans are prone to artifacts and poor resolution in proximity to the brainstem, whereas very early MRI may not show DAI or may definitely underestimate the evolving cascade of secondary damage. Furthermore, even when medical and surgical treatments are timely undertaken, 3cnP may not recover; in fact, fewer than 5% of cases eventually show a complete recovery [4–10].

To elucidate the mechanism of injury underlying 3cnP, we present five exemplificative cases of isolated 3cnP in patients suffering severe closed TBI with documented Grade III DAI [16]. Together with a literature review, these cases will help to outline four possible mechanisms of injury in mild to severe TBI and better explain the anatomical basis of 3cnP.

## 2. Case Presentations

**Case 1 .**

**Figure 1:**
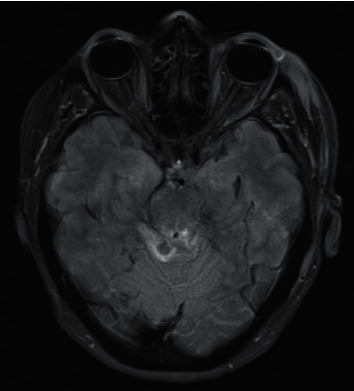
Axial FLAIR MRI showing Grade III DAI with hyperintense lesions at the level of the tectum of the midbrain, in the superior cerebellar parenchyma and the superior cerebellar peduncle.

A 50-year-old lady female cyclist fell over the handlebars of her bicycle at moderate speed and landed 8 ft away. Her Glasgow Coma Score (GCS) was 3 at the scene with fixed dilated pupils bilaterally (8 mm). Her GCS rapidly improved to 9, but the pupils remained fixed and dilated. A CT scan of her brain on arrival to our A&E department showed traumatic subarachnoid haemorrhage in the left superior cerebellar cistern, left perimesencephalic, and quadrigeminal plate cistern; it raised suspicion of possible DAI. There were no other significant injuries except for a right internal carotid artery (ICA) dissection at the cervical segment, for which she was started on aspirin. She was tubed and her initial ICP was 14 mmHg; hence, she received only medical treatment with deep sedation and optimisation of blood pressure to ensure adequate cerebral blood flow (CBF). On sedation hold, at 48 hours from the event, she showed improvement in her level of consciousness (GCS 10T) and was extubated after one day. However, her pupils remained unreactive and dilated at 8 mm bilaterally. Visual acuity was recorded as perception of hand movements only. She had a divergent gaze and was unable to move her gaze up or down, in keeping with bilateral oculomotor nerve dysfunction. MRI of her head (see Figure 1) showed Grade III DAI involving the tectum of the midbrain bilaterally, the inferior colliculi, and the superior cerebellar peduncles bilaterally. Although her visual acuity subsequently improved, there was very limited recovery in ocular movements. Although she had prolonged posttraumatic amnesia and short-term memory problems, she eventually made a good recovery and was discharged to rehab with a GCS of 14.

**Case 2 .**

**Figure 2:**
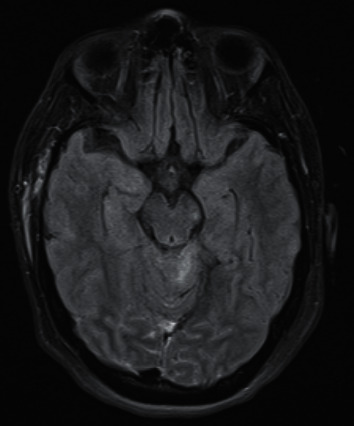
Axial FLAIR MRI, showing Grade III DAI with hyperintense lesions evident in the left midbrain and pons.

A 36-year-old female pedestrian was hit by a car. On scene, her GCS was 8 with a right-sided fixed and dilated pupil. She was noted to be moving all limbs. An initial CT head showed subarachnoid blood in the right sylvian fissure and overlying the right cerebral convexity and interpeduncular fossa. Extracranial injuries included a right tibial/fibular fracture and a pelvic fracture. She was extubated 2 days after admission; however, she continued to have a right 3cnP. She was mobilised and started early physiotherapy in the neurosurgical ward. Her MRI (see Figure 2) showed a Grade III DAI with involvement of the left side of the midbrain and pons in addition to the right mesial temporal lobe. Overall, she made a good recovery allowing a direct discharge home; nonetheless, her 3cnP had improved only slightly upon discharge.

**Case 3 .**

**Figure 3:**
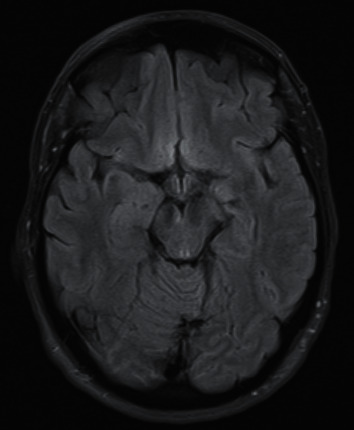
Axial FLAIR MRI showing injury in the medial aspect of the right cerebral peduncle close to the root entry zone of the right third nerve.

A 54-year-old female sustained a head injury upon falling from a horse. Her GCS was 15 on scene and she was moving all limbs but had a right fixed and dilated pupil with accompanying ptosis. Her eye was in the classic “down and out” position. An initial CT head showed subarachnoid haemorrhage in the anterior cingulate and right frontal sulci and interpeduncular fossa. She had no other injuries. A CT angiography indicated a dissected right petrous ICA with possible extension of the dissection flap through the right cavernous ICA into the supraclinoid ICA. There was also a small pseudoaneurysm arising from the right cavernous ICA. However, an MRI showed a small haemorrhagic contusion along the lateral aspect of the left side of the midbrain and a very localised hyperintense signal change in the medial aspect of the right cerebral peduncle close to the root entry zone of the right third cranial nerve (see Figure 3). This was interpreted as the cause for the 3cnP, and an MRA became necessary to rule out the possibility of a cavernous ICA dissection.

**Case 4 .**

**Figure 4:**
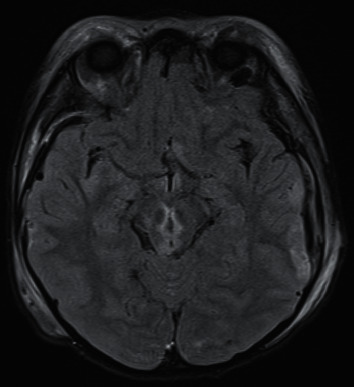
Axial FLAIR MRI showing multiple injuries in the tegmentum, tectum, and periaqueductal parenchyma of the midbrain, with involvement of the right cerebral peduncle, the right side of the pons, and the right middle cerebellar peduncle.

A 6-year-old boy was found by ambulance crew on GCS of 4 with fixed and dilated pupils following a head injury in which a concrete table fell on his head. Initial CT head imaging showed extensive bilateral calvarial, skull base, and facial fractures, bilateral subdural haematomas with minimal mass effect, multiple tiny intraparenchymal contusions within the frontal lobes and brainstem, and a small amount of intraventricular blood. Bilateral orbital subperiosteal haematomas were noted, but the globes and optic nerves were intact. He had an ICP bolt inserted, with opening pressures of 4 mmHg. A follow-on MRI (see Figure 4) showed a haemorrhagic shear injury affecting the tegmentum, tectum, and periaqueductal parenchyma of the midbrain, right cerebral peduncle, right side of the pons, right middle cerebellar peduncle, and superior aspect of the right cerebellar hemisphere. Management included prolonged intensive care unit stay (23 days) while he was kept deeply sedated and ventilated; during the admission, the left pupil regained a slight response to light, although the right one remained fixed and dilated. Following a long but sustained neurological recovery, he was back to full-time education without residual neurological deficits.

**Case 5 .**

**Figure 5:**
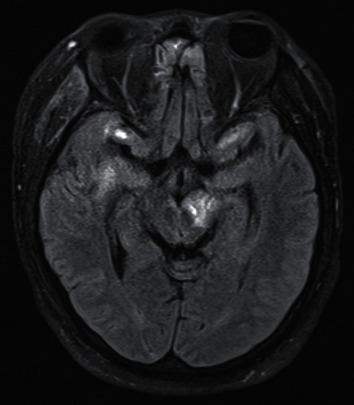
Axial FLAIR and sagittal T1WI MRI showing a significant injury to the left midbrain and multiple intraparenchymal contusions in the frontal, temporal, and parietal lobes.

A 37-year-old cyclist was hit by a car. He was confused with pupils equal and reactive on the scene; however, he rapidly decreased his level of consciousness during transfer to the A&E where he was in GCS of 5 with a right dilated and unreactive pupil. His CT showed extensive traumatic subarachnoid haemorrhage and contusions through both cerebral hemispheres. He had an ICP inserted, with opening pressures of 29 mmHg; this was managed with insertion of external ventricular drain. The MRI (see Figure 5) showed multiple haemorrhagic lesions involving the corpus callosum and brainstem consistent with DAI, a contusion in the left internal capsule and in the left frontal lobe. He eventually recovered to a GCS of 15, although he continued to have minimal cognitive impairment and decreased visual acuity in his right eye.

## 3. Discussion

The intracranial course of the oculomotor nerve runs from the anterior surface of the mesencephalon and advances between the superior cerebellar artery and the posterior cerebral artery. The third cranial nerve runs parallel, lateral, and below the posterior communicating artery (PCA), with the medial portion of the uncus lateral to it and enters the lateral wall of the cavernous sinus. It divides into its superior and inferior branches at the level of the superior orbital fissure. Along this course, fascicules are labelled as the subarachnoid segment, cavernous segment, orbital apex segment, and intraorbital segment [1, 4, 10, 17]. Once it reaches the orbit, it innervates the extraocular muscles of the eye: superior rectus, inferior rectus, medial rectus, inferior oblique, levator palpebrae superioris, the ciliary muscles, and the constrictor pupillae muscles of the iris. An oculomotor nerve palsy thus presents with a dilated pupil, ptosis, and infraducted and abducted eyeball (the classic down and out sign).

TBI are responsible for most 3cnP [1, 4–10, 17–23]; however, the incidence of isolated 3cnP not caused by space-occupying lesions such as posttraumatic subdural or extradural haematomas is rare. Multiple cranial nerve injuries tend to occur in the context of TBI, regardless of its severity. Our exemplificative cases provide a wide range of different presentations, showing that a 3cnP can be found at the scene or later on after admission to the hospital; it can be unilateral or bilateral and can be associated with other cranial nerve deficits.

The pathological mechanisms leading to isolated 3cnP can be direct or indirect. In patients with no obvious significant findings on CT, direct injury may result from extreme distraction of the nerve during the brain movement upon impact, rootlet avulsion, or distal fascicular damage. Direct injury can also occur as a result of compression, displacement, or deformity of the nerve by space-occupying lesions other than an obvious uncal herniation, such as a traumatic pseudoaneurysm [10, 23]. On the other hand, indirect injury can occur as a result of defective blood supply, caused by either the primary injury or its secondary metabolic cascade. Below we systematically explore four mechanisms of injury and subclassify them as either direct or indirect.

### 3.1. Mechanism 1: Shearing Injury (Direct Injury)

Balcer et al. [4] proposed proximal or distal fascicular damage with partial rootlet avulsion as common mechanisms of injury in traumatic 3cnP. This basically involves shearing injury to the nerve at his origin from the mesencephalon, which can be seen on MRI like a haemorrhage at the midbrain exit site of the oculomotor nerve. Gradient echo T2-weighted sequences are the most sensitive to detect haemorrhagic changes associated with shearing injury [4]. This mechanism of injury is generally seen in severe trauma and may be unilateral or bilateral as seen in Case 1.

### 3.2. Mechanism 2: Traction Injury (Direct Injury)

A possible second mechanism of 3cnP is nerve avulsion against the posterior petroclinoid ligament, where the nerve is stretched because of the downward brainstem displacement at the time of impact or herniation in the aftermath of TBI [8]. As such, this mechanism can explain a posttraumatic 3cnP from the outset or its late appearance in the subsequent hours such as in Cases 2 and 5.

The oculomotor nerve pierces the dura of the cavernous sinus through the oculomotor triangle. This triangle is bound by the anterior and posterior clinoid processes and the petrous apex. The medial margin of this triangle is formed by the interclinoid ligament, which extends from the anterior to the posterior clinoid process. The lateral margin is formed by the anterior petroclinoid ligament, which extends from the anterior clinoid process to the petrous apex, whereas the posterior margin is formed by the posterior petroclinoid ligament, which extends from the posterior clinoid process to the petrous apex. The oculomotor nerve runs over the posterior petroclinoid ligament. Direct injury to the pupillomotor fibres on the ventromedial surface of the nerve may occur when the nerve is stretched against this tough ligament as the brainstem moves downward at the time of impact. The nerve then becomes swollen, and ischemia could result from dural constriction at the point where the nerve pierces the dura of the cavernous sinus [21, 22].

### 3.3. Mechanism 3: Vascular Compression (Direct Injury)

Vascular compression was only briefly mentioned as a possible cause of oculomotor nerve damage in previous reports [10, 23]. The oculomotor nerve has an intimate relationship with the PCA and ICA, making the fibres, especially the pupillomotor ones, vulnerable to compression arising from a vascular anomaly. A painful oculomotor palsy is a well-established symptom and sign of an enlarging PCA aneurysm, and a traumatic pseudoaneurysm in this area may also cause neural compression (an oculomotor palsy). However, many patients suffering TBI are unconscious and so unable to report ocular pain.  Case 3 is a typical example of this rare scenario, which should always be kept into account as a fourth possible mechanism of injury whenever isolated palsies are noticed without involvement of the IV or VI nerves.

### 3.4. Mechanism 4: Defective Blood Supply (Indirect Injury)

Muthu and Pritty suggested that the III cranial nerves may suffer from disturbances in their blood supply or detrimental biochemical effects arising from the head injury [20]. The frontal, zygomatic, and maxillary bones (the facial “crumple zone”) frequently absorb the initial impact and are fractured in trauma. The III, IV, and VI cranial nerves and their supplying arteries are in close relationship to these bones, especially at the anterior portion of the cavernous sinus. The blunt movement and distortion of these bony structures may disrupt the vessels that provide the fragile pial blood supply to the nerves (1). To this regard, it is important to consider the blood supply to the oculomotor nerve: its proximal, extracavernous portion is supplied by perforators arising from the PCA, whereas its middle portion has no specific extraneural supply and its distal, intracavernous portion is supplied by perforators from the cavernous ICA. Therefore, any maxillofacial trauma such as in Case 4, any medial orbital fracture, and any dissection of the cavernous ICA can compromise the vascular supply of the distal nerve, causing a 3cnP.

### 3.5. Management and Prognosis

The prognosis of patients presenting with unilateral or bilateral fixed and dilated pupils in the setting of TBI without a mass lesion is generally devastating [24]. Decision-making in TBI should always be in adherence to internationally agreed management protocols relying on clinical history, neurological examination, neuroradiological investigations, and continuous multimodality monitoring [14, 16]. While the goal should be to maintain a good ICP and CBF, prognostic factors are to be kept into account [15, 24]. An isolated 3cnP has been for a long time considered as an end-stage sign in the pathological evolution of TBI. However, this sign may not always be a marker of irreversible diffuse brain injury and has the potential to confound early management and prognostication. Our exemplificative cases demonstrate that despite the long clinical course, recovery to a good neurological outcome is not impossible in both adult and paediatric patients.

A methodological approach to 3cnP is of paramount importance to ensure satisfactory outcomes. A baseline ophthalmology assessment should always be requested and initial clinical findings monitored over time until additional investigations could be carried out.

From a neuroradiology perspective, CT and MRI (particularly gradient echo and FLAIR sequences) should always be considered in patients with isolated 3cnP. Vascular imaging in the form of either CTA, MRI, or diagnostic digital selective angiography (DSA) should be requested whenever a vascular dissection is suspected. Interventional radiologists should be involved in the management of posttraumatic pseudoaneurysms, which might cause direct vascular compression [10, 23, 24].

## 4. Conclusion

Although isolated traumatic 3cnP is relatively uncommon in TBI, it may not represent a prognostic sign of devastating or unsurvivable injury. In all circumstances, it is important to correlate these signs with adequate imaging and additional investigations. Only a comprehensive assessment can enable clinicians and neurosurgeons to timely identify the underlying aetiology/mechanism of injury and provide their patients with adequate solutions.

## Data Availability

Data are available upon request to our Institutional Review Board.
